# **Prognostic factors in patients with clinical stage I nonseminoma**—**beyond lymphovascular invasion: a systematic review**

**DOI:** 10.1007/s00345-022-04063-7

**Published:** 2022-07-29

**Authors:** Friedemann Zengerling, Dirk Beyersdorff, Jonas Busch, Julia Heinzelbecker, David Pfister, Christian Ruf, Christian Winter, Peter Albers, Sabine Kliesch, Stefanie Schmidt

**Affiliations:** 1 Department of Urology and Pediatric Urology, University Hospital of Ulm, Albert-Einstein-Allee 23, 89081 Ulm, Germany; 2 UroEvidence at Deutsche Gesellschaft Für Urologie, Berlin, Germany; 3 Clinic and Polyclinic for Diagnostic and Interventional Radiology and Nuclear Medicine, University Hospital Hamburg-Eppendorf, Hamburg, Germany; 4 Department of Urology, Charité Universitaetsmedizin Berlin, Berlin, Germany; 5 Department of Urology and Paediatric Urology, Saarland University Medical Centre and Saarland University, Homburg, Germany; 6 Department of Urology, University Hospital Cologne, Cologne, Germany; 7 Department of Urology, Bundeswehrkrankenhaus, Ulm, Germany; 8 Urologie Neandertal, Erkrath, Germany; 9 Department of Urology, University Hospital Düsseldorf, Düsseldorf, Germany; 10 Centre of Reproductive Medicine and Andrology, Department of Clinical and Surgical Andrology, University Hospital Münster, Münster, Germany

**Keywords:** Nonseminoma, Recurrence, Lymphovascular invasion, Embryonal carcinoma, Prognostic factor, Prognosis

## Abstract

**Objective:**

To systematically evaluate evidence on prognostic factors for tumor recurrence in clinical stage I nonseminoma patients other than lymphovascular invasion (LVI).

**Methods:**

We performed a systematic literature search in the biomedical databases Medline (via Ovid) and Cochrane Central Register of Controlled Trials (search period January 2010 to February 2021) for full text publications in English and German language, reporting on retro- or prospectively assessed prognostic factors for tumor recurrence in patients with stage I nonseminomatous germ cell tumors.

**Results:**

Our literature search yielded eleven studies reporting on 20 potential prognostic factors. Results are based on cohort studies of mostly moderate to low quality. Five out of eight studies found a significant association of embryonal carcinoma (EC) in the primary tumor with relapse. Among the different risk definitions of embryonal carcinoma (presence, predominance, pure), presence of EC alone seems to be sufficient for prognostification. Interesting results were found for rete testis invasion, predominant yolk sac tumor, T-stage and history of cryptorchidism, but the sparse data situation does not justify their clinical use.

**Conclusions:**

No additional factors that meet the prognostic value of LVI, especially when determined by immunohistochemistry, could be identified through our systematic search. The presence of EC might serve as a second, subordinate prognostic factor for clinical use as the data situation is less abundant than the one of LVI. Further efforts are necessary to optimize the use of these two prognostic factors and to evaluate and validate further potential factors with promising preliminary data.

**Supplementary Information:**

The online version contains supplementary material available at 10.1007/s00345-022-04063-7.

## Introduction

Testicular germ cell cancer is the most common malignancy in young men and its initial treatment consists of inguinal orchiectomy. Pathohistologic examination of the affected testis reveals nonseminoma in about 40% of the cases [1]. Upon radiographic assessment about 60% of the diagnosed nonseminomas exhibit no tumor-suspicious abnormalities and are, therefore, classified as clinical stage I [1]. Data from a meta-analysis including 7113 patients revealed that despite the absence of pathologic findings at the initial staging, about 18.6–41.3% of stage I nonseminoma patients managed by surveillance develop tumor recurrence during follow-up.

Established adjuvant treatment options to reduce the risk of relapse are adjuvant chemotherapy with one cycle of bleomycin, etoposide and cisplatin (BEP) or nerve-sparing modified retroperitoneal lymphadenectomy (nsRPLND) [3].

Adjuvant chemotherapy with 1 × BEP is highly effective in this situation as it results in a significant reduction of the relapse rate to ≤ 3% for 1 × BEP [4]. Possible drawbacks of 1xBEP are short-term complications as neutropenic fever, thromboembolism and reduced fertility as well as long-term toxicities with a possible decrease in relative survival manifesting after decades [5].

Another option, which is recommended only in very selected cases, is nsRPLND, which achieves an almost complete local control without the application of adjuvant chemotherapy in pathohistological stage I and IIA disease, but it is hampered by a 10.2% rate of metastasis developing outside the retroperitoneum [6].

The third option for nonseminoma stage I patients is Surveillance, which is associated with a high cure rate of > 99% [7]. However, about 15–45% of patients relapse during Surveillance with the need of an induction chemotherapy with at least 3 cycles of BEP [8].

In summary, all three approaches are characterized by an approximately 50–85% risk for overtreatment (for 1 × BEP or nsRPLND) or 15–50% risk for undertreatment (for Surveillance), leading to unnecessary adjuvant treatments in the former and to a higher burden of chemotherapy in the latter case.

To meet this issue, individualizing initial treatment for nonseminoma stage I patients by risk-adapted treatment approaches has been intensively studied during the last decades. A number of clinical trials has, therefore, focused on the identification of prognostic factors to stratify patients according to their relapse risk. The best validated prognostic factor in this context is lymphovascular invasion (LVI), i.e. infiltration of tumor cells into lymphatic or vascular structures within the primary [2, 9]. The application of LVI allows to form two risk cohorts, segregating the overall 25–30% relapse risk of nonseminoma stage I patients to high-risk group (LVI +) with a ~ 47.5% relapse risk and a low-risk group (LVI-) with a ~ 16.9% relapse risk [2]. LVI status can be implemented into a treatment algorithm that consists of 1 × BEP for the LVI + group and Surveillance for the LVI- group. This results in a significant advantage according to the necessary treatment burden to achieve cure in a larger collective of patients. Application of this risk-adapted approach results on average in 0.41 less cycles BEP per patient in the LVI + group and of 0.61 less cycles BEP per patient in the LVI- group, when compared with the respective alternative treatment option [10].

Consequent use of this broadly accepted risk-adapted approach is still far from an ideal state for tailored therapy in this young patient group. Using this approach still results in a about 50% risk of overtreatment for LVI + patients and in an ~ 15% risk of undertreatment for LVI- patients. Therefore, further effort is necessary to identify additional prognostic factors that might outperform or supplement LVI in the prognostication of relapse in non-semimoma stage I patients.

The aim of the present work is to review recent evidence the current knowledge on possible additional prognostic factors for non-semimoma stage I, which could help to optimize the decision on adjuvant treatment for these patients.

## Methods

This work is based on a former systematic literature search that was conducted for the elaboration of the first German clinical practice guideline [11, 12]. In this context, several systematic literature searches were conducted. We present here the results combined with an update search on this topic.

### Systematic literature search

We performed a systematic literature review in accordance to the preferred reporting items for systematic reviews and meta-analyses (PRISMA) guidelines [13]. Our search was limited to full text publications in English and German language published in the biomedical databases Medline (via Ovid) and Cochrane Central Register of Controlled Trials (search period January 2010 to February 2021). We considered randomized clinical trials and prospective and retrospective observational comparative studies. Case reports, case series, editorials, comments and conference abstracts were excluded. An additional search for unpublished data and ongoing studies was conducted in clinical trial registers (clinicaltrials.gov/ and www.who.int/ictrp/). We have contacted the study coordinators in case of missing information for studies identified in the trial registries. We also hand-searched the reference lists of included studies to determine additional, potentially relevant studies.

Studies were considered if they included patients (≥ 18 years) with initial stage I nonseminomatous germ cell tumors that were managed either by surveillance or by adjuvant therapies such as nsRPLND or 1–2 cycles of BEP chemotherapy. Studies had to report on prognostic markers for tumor recurrence, either as single marker or a combination of markers. As vascular invasion (VI) is an established prognostic marker for patients with stage I nonseminomatous germ cell tumors, our search focused on prognostic markers other than VI. Studies on seminomas only or other than stage I disease stages were excluded. Studies published between 2010 and 2021 in English or German language were considered.

### Literature screening, data extraction and quality assessment

One review author screened the titles and abstracts and afterwards the full texts of the retrieved references and determined the relevance for inclusion. For included studies, one author extracted relevant data in evidence tables. The study quality was appraised by one author using the QUIPS tool for the risk of bias assessment in prognostic studies [14] and the level of evidence was rated according to the Oxford criteria [15]. In any case of uncertainty, another review author was involved in each of the above-mentioned steps and a consensus was reached by discussion.

## Results

The literature search identified in total 2829 records (Supplemental Fig. 1). Eleven prognostic cohort studies, reporting a total of 2829 nonseminomatous germ cell tumor stage I (NSGCT I) patients, met our inclusion criteria (Supplemental Table 1). Study sample sizes ranged from 27 to 1226 NSGCT I patients. Further details on the study characteristics are summarized in Supplemental Table 1.

**Table 1:** Association of commonly reported features of the primary tumorwith time to relapse or relapse

LVI determination by immunohistochemistry (Vascular invasion scoring (D2–40 + FVIII + CD31))	Compared to H&E staining:	
	- Improved sensitivity (71.0% vs. 61.3%) - Decreased specifity (71.4 vs. 85.7%) - Identical accuracy (71.2% vs. 71.2%)	Lobo et al. 2019 [18]
Yolk Sac Tumor	Presence of Yolk Sac Tumor:	
	- Not significant on univariate analysis	Shinoda et al. 2018 [24]
	Predominant Yolk Sac Tumor:	
	- **OR 3.537 (1.076–11.628); *p* = 0.038**	Li et al. 2015 [22]
Teratoma mature/immature	Presence of Teratoma:	
	- OR 1.1 (0.5–2.4); *p* = 0.891	Nicolai et a. 2010 [23]
	Predominant Mature Teratoma:	
	- OR 0.400 (0.104–1.544); *p* = 0.184	Li et al. 2015 [22]
	Predominant Immature Teratoma:	
	- OR 0.824 (0.086–7.872); *p* = 0.866	Li et al. 2015 [22]
Seminoma	Predominant Seminoma:	
	- OR 3.471 (0.206–58.449); *p* = 0.388	Li et al. 2015 [22]
Rete testis invasion (RTI)	Presence of RTI:	
	- **HR 1.30 (1.02**–**1.69); *p* = 0.035**	Daugaard et al. 2014 [8]
T stage	T1 vs. T2 vs. T3:	
	- OR 1.041 (0.384–2.821); *p* = 0.937	Li et al. 2015 [22]
	T1 vs. T2/T3:	
	- **OR 4.4 (1.7–11.7); *p* = 0.003**	Nicolai et a. 2010 [23]
	- Not significant on univariate analysis	Shinoda et al. 2018 [24]
Primary tumor size	Tumor size < 4 cm vs. ≥ 4 cm:	
	- OR 2.055 (0.700–6.030); *p* = 0.190	Li et al. 2015 [22]

Significant results are highlighted in bold

A total of 20 different pathohistological, clinical and molecular factors were reported (Table 1, Table 2, Table 3). Most of the factors (*n* = 7) were basic histopathologic characteristics of the primary tumor, including histological subtypes, features of differential pathology, tumor size, T-stage or rete testis invasion (RTI). Another three factors were also assessed on primary tumor material using immunohistochemistry or gene analysis. Out of ten clinical factors, four were of demographic (medical history), three were of serological (blood sample results) and another three were of radiologic nature.

**Table 2:** Association of Embryonal Carcinoma (multivariate analysis) with time to relapse or relapse

Risk factor	Study results	Reference
Embryonal Carcinoma (EC)	Presence of EC:	
	- **HR 2.73 (1.94**–**3.85); *p* = 0.0013**	Daugaard et al. 2014 [8]
	- Stratified log rank p value 0.243	Gilbert et al. 2016 [20]
	- **HR 10.239 (1.336–78.488); *p* = 0.0252**	Shinoda et al. 2018 [24]
	- Not significant on multivariate analysis	Sturgeon et al. 2011 [25]
	Predominant EC:	
	- OR 0.71 (0.14–3.67) *p* = 0.69	Dong et al. 2013 [19]
	- OR 1.63 (0.37–7.09) *p* = 0.52	Howard et al. 2014 [21]
	- OR 1.133 (0.272–4.726) *p* = 0.864	Li et al. 2015 [22]
	- Not significant on multivariate analysis	Sturgeon et al. 2011 [25]
	Pure EC:	
	- **HR: 1.74 (1.10–2.74) *p* = 0.02**	Sturgeon et al. 2011 [25]
	Categorized (≤ 25% vs. 26–99% vs. 100%):	
	- **Stratified log rank *p* value 0.006**	Gilbert et al. 2016 [20]
	Continuous (%EC values):	
	- **OR 3.5 (1.4–9.0)**; increased risk up to ~ 50% and decreased risk for higher %EC values	Nicolai et al. 2010 [23]

Odds and hazard ratios were derived from multivariate analyses if not otherwise indicated. Significant results are highlighted in bold

**Table 3:** Association of serum tumor markers, patient characteristics and imaging features with time to relapse or relapse

Preoperative AFP levels	Normal AFP vs. elevated AFP:	
	- 1.160 (0.356–3.785) 0.806	Li et al. 2015 [22]
	AFP level ≤ 80 ng/ml vs. > 80 ng/ml:	
	- Not significant on univariate analysis	Shinoda et al. 2018 [24]
Preoperative HCG levels (normal vs. elevated)	Normal HCG vs. elevated HCG:	
	- OR 1.222 (0.426–3.509); *p* = 0.709	Li et al. 2015 [22]
Preoperative LDH levels (normal vs. elevated)	Normal LDH vs. elevated LDH:	
	- Not significant on univariate analysis	Shinoda et al. 2018 [24]
CXCL12 expression	CXCL12 staining absent/weak vs. moderate/high:	
	- **Stratified log rank *p* value 0.009**	Gilbert et al. 2016 [20]
Gene set enrichment analysis	10-probe gene signature:	
	- Not significant; *p* = 0.36	Lewin et al. 2018 [35]
	30-probe gene signature:	
	- Not significant; *p* = 0.31	Lewin et al. 2018 [35]
	100-probe gene signature:	
	- Not significant; *p* = 0.36	Lewin et al. 2018 [35]
Cryptorchidism	History of cryptorchidism vs. no history of cryptorchidism:	
	- **OR 0.07 (0.01–0.34); *p* = 0.001**	Dong et al. 2013 [19]
Laterality	- OR 1.62 (0.42–6.23); *p* = 0.48	Dong et al. 2013 [19]
Age	Age (continuous):	
	- OR 1.01 (0.95–1.05); *p* = 0.96	Dong et al. 2013 [19]
	Age (≤ 30 years vs. > 30 years):	
	- Not significant on univariate analysis	Shinoda et al. 2018 [24]
Obesity (BMI < 25 kg/m2 vs. BMI ≥ 25 kg/m2)	BMI ≥ 25 kg/m2 vs. BMI < 25 kg/m2:	
	- HR 0.78 (0.34–1.81); *p* = 0.57	McGregor 2019 [33]
Retroperitoneal lymph node configuration	Craniocaudal node length (continuous):	
	- **OR 1.15 (1.01, 1.31); *p* = 0.03**	Howard et al. 2014 [21]
	Nodal volume (continuous):	
	- OR 0.78 (0.21, 2.96); *p* = 0.72	Howard et al. 2014 [21]
	Short axis diameter (continuous):	
	- OR 1.18 (0.88, 1.59); *p* = 0.27	Howard et al. 2014 [21]

Odds and hazard ratios were derived from multivariate analyses if not otherwise indicated. Significant results are highlighted in bold

The quality of the included studies can be depicted from Supplemental Table 1. Risk of bias ranged from low (5 studies) and moderate (4 studies) to high (2 studies), partly lowering the confidence in the presented results for the latter one. Studies with high risk of bias presented with insufficient information on the definition of prognostic factor or outcome, no reporting of dropouts during the study period or missing adjustment of potential confounders.

Despite many histopathological, clinical or molecular factors were identified by our literature search, most of the data on each of the factors rely on only one or two mostly small retrospective studies. The presence of embryonal carcinoma forms an exemption from this, as it was investigated by a total of eight studies included in our review. In the following section the most relevant prognostic factors retrieved by our search will be discussed more in detail.

## Discussion

### Reflecting on the precision of LVI assessment

LVI is the best validated prognostic factor for relapse in nonseminoma stage I patients, although its far away from an optimal prognosticator, as its sensitivity and specifity do not exceed 75% [2, 9]. However, significant disagreement between peripheral, general pathologies and pathology departments with more expertise in testicular germ cell tumor assessment and a high rate of interobserver variability using conventional hematoxylin & eosin (H&E) staining was reported [16, 17]. A recent study from the Netherlands, that was retrieved from our search, investigated the value of CD31, FVIII, and D2–40 immunohistochemistry for LVI assessment with 52 nonseminoma stage I patients undergoing surveillance [18]. Compared to H&E, the use of immunohistological markers resulted in improved sensitivity (71.0 vs. 61.3%) on the cost of decreased specificity (71.4 vs. 85.7%) and with identical accuracy (71.2 vs. 71.2%) (Table 1). This was clinically relevant, as after a median follow-up of 66 months three of the eight more cases detected by IHC staining developed relapse and the two cases in which LVI was reclassified to be absent on IHC did not develop relapse. Another interesting finding of this study was, that consideration of the origin of LVI positivity, differentiating between lymph vessel infiltration only (L1, V0), blood vessel infiltration only (L0, V1) and presence of both (L1, V1) had a high impact on the prediction of relapse [18]. The authors found, that all eight patients with “double-vascular invasion” (L1, V1) experienced disease recurrence. This interesting finding of improved prediction with consideration of “double-vascular invasion” has not yet been validated in a larger series.

### Features of the primary tumor as prognostic factors

The presence of embryonal carcinoma (EC) is associated to LVI, as it is the most frequent tumor component that can be found within the blood or lymph vessels upon pathohistological examination [18]. Independent from the capacity to invade vessels, the role of EC tissue in the primary tumor for prognostification of disease relapse was investigated by eight studies retrieved from our literature search [8, 19–25](Table 2). Five of these studies found a significant correlation of EC with disease relapse [8, 20, 23–25]. Interestingly, the presence of EC alone was more reliantly associated with relapse than the presence of predominant EC (i.e. > 50% EC) in this analysis, as this was true for two out of four studies for the former and for none out of four studies for the latter definition. The largest study of our literature search by Daugaard et al. only differentiated absence or presence of EC without quantitative pathology assessment using a definite cutoff, for example, 50% [8]. This study revealed an around three fold higher risk for relapse for patients with the evidence of EC in their primary tumor [hazard ratio 2.73 (95% confidence interval (95% CI) 1.94–3.85)] [8]. The predictive value was even higher, when presence of EC was combined with LVI and RTI, reaching a hazard ratio of 5.65 (95% CI 3.74–8.53) with a 5-year relapse rate (5-year RR) of 50%. Of note, LVI alone had a surprisingly low predictive value with a hazard ratio of 1.57 (95% CI 1.22–2.02) and a 5 year RR of 18% in this large cohort, possibly affected by the known difficulties in reliable LVI assessment [18].

It is known, that LVI is positively correlated with rising EC proportions and inversely correlated with higher amounts of teratoma in the pathological specimen [26]. As the presence of EC and the presence of LVI seem to be interconnected, this might raise the question, if LVI alone might be sufficient for the prediction of relapse. In all of the five positive studies the presence of EC was prognostic for relapse independent from LVI upon multivariate analysis. This largely rules out a plain association of the presence of EC with the established prognostic factor LVI and underlines the independent value of EC in this context.

Different tumor components of nonseminomas as well as their quantitative evaluation was not only investigated for EC, but also for other tumor differentiations. The most comprehensive analysis on tumor histologies was conducted by a Chinese study, comprising 78 stage I nonseminomas [22]. Interestingly, in contrast to teratoma or seminoma, predominant yolk sac tumor (pYST) was strongly associated with relapse in their cohort. Ten out of 26 patients with pYST had a relapse during follow-up, whereas this was true only for 8 out of 52 patients without pYST, resulting in a considerable hazard ratio of 3.54 (95% CI 1.08–11.63) in multivariate analysis. This encouraging result is conflicting with data from another study included in our analysis, where the presence (not the predominance) of YST showed no correlation with relapse [24]. To what extent the percentage of YST might play a role in this context remains speculative, as the authors of the latter study did not perform quantitative pathology and we found no further data on this.

For rete testis invasion, a prognostic factor, which was extensively studied for stage I seminoma, our literature search yielded only one study for stage I nonseminoma [8, 27]. The large Danish cohort study found a weak but significant association of rete testis invasion with tumor recurrence with a hazard ratio of 1.30 (95% CI 1.02–1.69) [8].

For TNM T-stage, which is a composite of local tumor extension to neighbouring macroscopic structures (e.g., tunica albuginea or epididymis) as well as microscopic infiltration of blood or lymph vessels (corresponding to LVI), data from three studies were included in our report [22–24]. Two studies with a relatively small sample size found no association of T-stage with relapse [22, 24]. In contrast, a large series of 322 nonseminoma stage I patients revealed a strong correlation of a ≥ 2 T-stage with relapse after staging RPLND with an odds ratio of 4.4 (95% CI 1.7–11.7) *p* = 0.003 [23]. This result supports the EAU guideline recommendation, where a ≥ 2 T-stage and not the presence of LVI alone is used as a decision tool for or against adjuvant chemotherapy with 1 × BEP [3].

Size of the primary tumor, continuous or with a cutoff of 4 cm, which is the best described and well accepted prognostic factor for recurrence in stage I seminoma [27], is for nonseminoma patients only studied by Li et al. with data based on a circumscript sample of 78 patients only [22]. They found a non-significant trend towards a higher risk of recurrence for larger tumors with an odds ratio of 2.055 (95% CI 0.700–6.030). In a former large series from 2007, that comprised 779 patients, primary tumor size was also not predictive of occult metastatic disease (*p* = 0.167) [28]. This is supported by a recently published systematic review, in which tumor size was not predictive for recurrence in 5 out of 6 studies [2].

### Serum tumor markers as prognostic factors

Serum tumor markers occupy a special position for the management of TGCT patients. Elevated pre-orchiectomy tumor markers support the suspicion of a TGCT, constantly or rising post-orchiectomy markers suggest metastatic disease even in the absence of suspect findings on cross-sectional imaging. Hypothetically, high pre-orchiectomy marker levels might be able to predict recurrence in stage I nonseminoma patients, although they initially decline to normal after surgery (Table 3). Two studies, reporting on preoperative HCG, AFP and LDH levels were identified by our search [22, 24]. None of the mentioned serum tumor markers were associated with tumor recurrence in their analyses, whereby the number of included patients was limited.

MiR-371a-3p (M371), a promising novel tumor marker, which showed high correlation with the presence of a germ cell tumor, was recently studied in a cohort of 151 stage I TGCT patients (101 seminomas, 50 nonseminomas) undergoing surveillance [29, 30]. Contrary to the expectations, this study found no association of post-orchiectomy M371 levels with relapse for seminoma patients (OR 1.3, 95% CI 0.87–2.02) as well as for nonseminoma patients (OR 0.45, 95% CI 0.21–1.00) [29]. If methodological differences, for example, the timepoints of postoperative M371 determinations, between the present study and original report of Dieckmann et al. might have played a role, is under debate. Two prospective studies, that should answer this question and help to elucidate the role of M371 as a predictive marker for occult or early metastasis, are currently recruiting (NCT04435756; GTCSG M371 follow-up trial).

### Patient characteristics as prognostic factor

A prospective study from China comprising 89 patients had its focus on patient characteristics apart from histological features [19]. First, age as a continuous variable was reported to have no association with disease relapse, in line with another study that used a cutoff of 30 years [24]. Whereas laterality had no predictive value, history of cryptorchidism was strongly correlated with relapse [19]. In the surveillance group (*n* = 38), patients with history of cryptorchidism had a higher relapse rate than those without cryptorchidism (50 vs. 13.3%, *p* = 0.02). Cryptorchidism is part of the testicular dysgenesis syndrome (TDS), which also comprises hypospadia and semen parameters disorders. Selvi et al. found a strong correlation of TDS with the development of metastasis for TGCTs [31]. These results on cryptorchidism and TDS have to be interpreted with care, as the results are based on a small patient cohort and the characteristics of cryptorchidism as ipsi- or contralaterality and treatment history were not defined.

Obesity at the time of diagnosis has been associated with better outcomes in some metastatic cancers, such as renal-cell cancer [32]. The study of McGregor et al. focused on the association of obesity with outcomes in TGCT patients [33]. A high body mass index, dichotomized at 25 kg/m^2^, was not correlated with relapse in stage I nonseminoma patients (hazard ratio 0.78 (95% CI 0.34–1.81)).

### Imaging features as prognostic factor

Howard et al. investigated the prognostic role of retroperitoneal lymph nodes smaller than 10 mm at the initial staging computed tomography [21]. The radiological parameters greatest short-axis node diameter (OR 1.18 (95% IC 0.88–1.59)) and nodal volume (OR 0.78 (95% CI 0.21–2.96)) were not correlated with tumor recurrence. Interestingly, craniocaudal node length was predictive for relapse in a significant but not clinically meaningful dimension (OR 1.15 (1.01–1.31)). A more sophisticated imaging to interpret data from computed tomographies beyond size measurements is radiomics. Radiomics uses data from the internal texture of lymph nodes, which help to train a diagnostic software-based algorithm for discriminating benign from malignant tissue. This technique has so far been only applied to lymph nodes larger than 10 mm (clinical stage ≥ IIA) for the assessment of post-chemotherapeutic residual masses and still requires testing for subclinical enlarged lymph nodes in stage I patients [34, 35].

### Implications for further research

Future studies might concentrate towards an improved use and assessment of the existing prognostic factor LVI. Assessment of LVI should by now rely on an additional immunohistochemistry assessment with specific antibodies, such as CD31, FVIII, and D2–40 [18]. This improves the reliability of LVI assessment without a relevant increase in time or cost. Furthermore, a more differentiated view on LVI, which takes into account the invasive tumor component (EC or others) as well as the affected vessels (L1V0, L0V1, L1V1) is a promising approach to pull out more from LVI as to date.

Among the plethora of predictive factors presented in this work, history of cryptorchidism sticks out, as it had a very strong correlation with relapse in stage I nonseminoma patients [19]. As this was only investigated in one small study with a low event rate (*n* = 8), confirmative analyzes are necessary to further evaluate this interesting finding.

## Summary

**Fig. 1:**
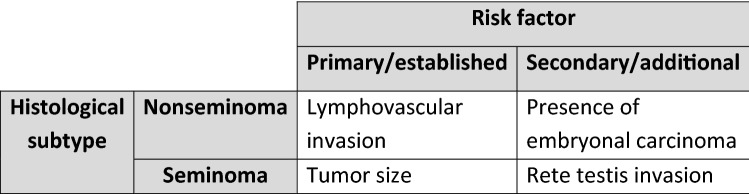
Risk factors for relapse in clinical stage I testicular cancer

No additional prognostic factors that reach a similar prognostic value compared to LVI could be identified through our search. The presence of EC, a well described prognostic factor from previous reports, has some additional predictive value, although smaller than that one of LVI (see Fig. 1). In line with a recent meta-analysis, the trials included in our work were not able to define a cutoff for a necessary percentage of EC within the primary tumor. The information on the presence or absence of EC can be integrated into the decision-making on the necessity of adjuvant treatment in stage I nonseminoma, which is still mainly driven by a reliably assessed LVI status.

## Supplementary Information

Below is the link to the electronic supplementary material.

Supplementary file1 (DOCX 34 kb)

Supplementary file2 (DOCX 29 kb)
