# Supplementary material for: Prognostic factors in patients with clinical stage I nonseminoma—beyond lymphovascular invasion: a systematic review
Source: World J Urol. 2022 Jul 29;40(12):2879–87. doi: 10.1007/s00345-022-04063-7 (PMC9712284; doi:10.1007/s00345-022-04063-7)
Supplement: Supplementary file 1 — Supplementary file1 (DOCX 34 kb) [file 345_2022_4063_MOESM1_ESM.docx]

**Supplements**

**Supplemental Figure 1: PRISMA Flow chart**

Records excluded
(n = 2531)

Records after title & abstract screening
(n = 64)

Records after duplicates removed
(n = 2731)

Records identified through database searching
(n = 2829)

Identification

Screening

Eligibility

Full-text articles excluded
(n = 8)

Main reasons for exclusion:

- wrong prognostic factor

- mixed patient population

Full-text articles assessed for eligibility
(n = 19)

Included

Studies included in qualitative synthesis
(n = 11)
