# Supplementary material for: Prognostic factors in patients with clinical stage I nonseminoma—beyond lymphovascular invasion: a systematic review
Source: World J Urol. 2022 Jul 29;40(12):2879–87. doi: 10.1007/s00345-022-04063-7 (PMC9712284; doi:10.1007/s00345-022-04063-7)
Supplement: Supplementary file 2 — Supplementary file2 (DOCX 29 kb) [file 345_2022_4063_MOESM2_ESM.docx]

**Supplemental table 1: Characteristics of included studies**

| Reference | Design  Country  Inclusion period  Follow-up | Number of patients | Initial treatment | Investigated prognostic factor(s) | Primary Endpoint | Statistical analysis | Results | Level of evidence  Risk of bias  *additional comments* |
| --- | --- | --- | --- | --- | --- | --- | --- | --- |
| Daugaard  2014 | prognostic cohort study  Denmark  1984-2007  Median follow-up: 180 mo (1-346 mo) | 1226 | Surveillance | Vascular invasion  present  Rete testis  invasion  present  Embryonal  carcinoma | Relapse-Free Survival | Univariable und multivariable analysis | **Multivariable analysis**  Vascular invasion present: HR 2.20 (95% CI 1.64 - 2.99) p=0.001  Rete testis invasion: HR 1.47 (95% CI 1.10 - 1.98) p=0.010  Embryonal carcinoma: HR 3.85 (95% CI 2.03 - 7.32) p=0.001 | LoE 2b  RoB: low |
| Dong  2013 | prognostic cohort study  China  January 1997 - December 2009  Median follow-up  Surveillance: 60 mo (12–129 mo)  RPLND: 58 mo (13–102 mo)  Chemotherapy: 81 mo (13–175 mo) | 89 | Surveillance OR RPLND OR adjuvant chemotherapy  following orchiectomy | Treatment options  History of cryptorchidism  predominant embryonal carcinoma  Lymphatic/vascular invasion  Age  Laterality | Relapse | Multivariate analysis | **All patients**  Treatment options  OR 0.22 (0.06–0.92) p=0.04  History of cryptorchidism  OR 0.07 (0.01–0.34) p= 0.001  Laterality  OR 1.62 (0.42–6.23) p=0.48  Age  OR 1.01 (0.95–1.05) p=0.96  Predominant embryonal carcinoma  OR 0.71 (0.14–3.67) p=0.69  Lymphatic/vascular invasion  OR 5.02 (1.17–21.62) p=0.02  **Surveillance patients**  History of cryptorchidism  OR 0.09 (0.01–0.56) p=0.01  Laterality  OR 0.97 (0.19–4.84) p=0.97  Age  OR 1.16 (0.78–2.01) p=0.05  Predominat embryonal carcinoma  OR 1.67 (0.26–10.77) p=0.06  Lymphatic/vascular invasion  OR 12.10 (2.56–18.42) p=0.01 | LoE 2b  RoB: moderate  no clear definition of relapse (e.g. cut-off for serum tumor marker abnormally), no univariate analysis, dropouts not reported |
| Gilbert  2016 | prognostic cohort study  United Kingdom, Canada  Median follow-up: 48 mo (2-84 mo) | 190 | Surveillance | CXCL12 expression  Embryonal carcinoma  VascuIar invasion | Risk of relapse | multivariate analysis | **Model 1**  VascuIar invasion (present vs absent): HR 3.29 (95% CI 1.68. 6.40) p<0.001  Embryonal carcinoma (continuous): HR 1.01 (95% CI 1.00 - 1.01)  p=0.012  **Model 2**  VascuIar invasion (present vs absent): HR 3.28 (95% CI 1.68 - 6.38) p<0.001  Embryonal carcinoma ≤25%: HR 1 (reference)  Embryonal carcinoma 26-99%: HR 1.67 (95% CI 0.73 -3.83)  p=0.019  Embryonal carcinoma 100%: HR 3.11 (95% CI 1.39 - 6.98)  **Model 3**  VascuIar invasion (present/absent): HR 4.33 (95% CI 2.23 - 8.40)  p=0.001  CXCL12 (absent/weak vs moderate/high): HR 0.43 (95% CI 0.22 - 0.86)  p=0.01 | LoE 4*  RoB: high  no period of recruitment described, prognostic factor is described but not extensively defined, dropouts not reported, no potential confounders reported (e.g. age) |
| Howard 2014 | prognostic cohort study  USA  1997 – 2010  follow-up:  at least 2 years | 62 (of 118) | Surveillance | craniocaudal  nodal length  embryonal  predominance  lymphovascular invasion  nodal volume  greatest short-axis-diameter | risk of relapse | multivariable models | **n=62 NSGCT patients**  Craniocaudal nodal length (cm): OR 1.15 (1.01, 1.31) p=0.03  Craniocaudal nodal length (Estimates for every 3-mm increase): OR 1.52 (1.03, 2.25) p=0.03  Embryonal-predominant (no=reference): OR 1.63 (0.37, 7.09) p=0.52  lymphovascular invasion (no=reference): OR 8.67 (1.38, 54.37)  p=0.02  Nodal volume (cm3):  OR 0.78 (0.21, 2.96) p=0.72  Greatest short-axis-diameter (mm): OR 1.18 (0.88, 1.59) p=0.27 | LoE 2b  RoB: low  *results only shown for patients with NSGCT* |
| Lewin  2018 | prognostic cohort study  USA  2000-2012 | 27 (of 57) | Surveillance | RNA extraction and gene expression analysis | Relapse | Wilcoxon test | **Relapsed vs. non-relapsed**  Vascular invasion  nonseminoma: 5/12 vs. 0/15  Median size  seminoma: 4 cm vs. 2.8 cm  **Relapsed**  Gene-set enrichment analysis revealed enrichment in pathways associated with differentiation, such as skeletal development (i.e. FGFR1, BMP4, GLI2, SPARC, COL2A1), tissue (i.e. BMP4, SPARC, COL13A1) and bone remodelling (i.e. CARTPT, GLI2, MGP).  A discriminating signature for relapsed and non-relapsed clinical stage I TGCTs using 10 and 30 gene sets was identified, but this was not confirmed when SGCTs and NSGCTs were analysed separately. | LoE 4*  RoB: high  baseline characteristics of the selected cases are not extensively described, potential confounders are not described and included in the statistical analysis, selective reporting of results for prognostic factors |
| Li  2015 | prognostic cohort study  China  1999 – 2013  Median follow-up time: 6.2 y (1–15 y) | 78 (of 163) | Surveillance | Lymphovascular invasion  Predominant presence of yolk sac tumor  Predominant presence of embryonal carcinoma  Predominant presence of mature teratoma  Predominant presence of immature teratoma  Predominant presence of seminoma  Age (≦30 vs. >30)  T classification (T1 vs. T2 vs. T3)  Preoperative AFP levels (normal vs. elevated)  Preoperative HCG levels (normal vs. elevated)  Primary tumor size (<4 cm vs. ≥4 cm) | Relapse-free survival | multivariate analysis | **Relapse-Free Survival**  Lymph vascular invasion  OR 6.521; 95 % CI 1.872–22.721 (p = 0.003)  Predominant presence of yolk sac tumor  OR 3.537; 95 % CI 1.076–11.628 (p = 0.038) | LoE 2b  RoB: low |
| Lobo  2019 | prognostic cohort study  Netherlands  1993-2006  Median follow-up: 66 mo (40-93 mo) | 52 | Surveillance | vascular invasion scoring  Immunohistochemistry (D2-40+FVIII+CD31) | relapse | Cohen κ  χ2 (with continuity correction)  Mann-Whitney U test  Kaplan-Meier estimator, log-rank test  Cox regression models | **Performance of Vascular Invasion Scoring in Predicting Disease Relapse**  Sensitivity=61.3%  Specificity=85.7%  Positive predictive value=86.4%  Negative predictive value=60.0%  Accuracy=71.2%  Adding immunohistochemistry (D2-40+FVIII+CD31):  Sensitivity=71%  Specificity=71.4%  Positive predictive value=78.6%  Negative predictive value=62.5%  Accuracy=71.2%  Multivariable analysis:  Vascular invasion was in independent predictor of relapse (HR 3.163; 95% CI 1.31-7.63). | LoE 2b  RoB: moderate  no relapse definition, unclear, how confounders were considered in the statistical analysis |
| McGregor  2019 | prognostic cohort study  USA  1997-2012  end of follow-up: July 31, 2014 | 280 (of 1161) | Surveillance OR RPLND OR adjuvant chemotherapy  following orchiectomy | Obesity  BMI < 25 kg/m2 vs. BMI ≥ 25 kg/m2 | relapse | logistic regression analysis  Cox regression models | **BMI**  Normal  no relapse: 142 (81%)  relapse: 34 (19%)  Overweight/Obese  no relapse: 308 (85%)  relapse: 55 (15%)  p=0.22  **Association Between BMI and Risk of Relapse**  All Patients (n= 539)  Overweight/Obese: HR 0.83 (95% CI 0.53-1.30) p=0.41  Among surveillance patients (n=293)  Overweight/Obese: HR 0.83 (95% CI 0.49-1.41) p=0.50  Among seminoma patients (n=259)  Overweight/Obese: HR 0.78 (95% CI 0.34-1.81) p=0.57  Among nonseminoma patients (n=280)  Overweight/Obese: HR 0.84 (95% CI 0.48-1.46) p=0.57 | LoE 2b  RoB: low  no relapse definition |
| Nicolai  2010 | prognostic cohort study  Italien  1985 – 1995  Median follow-up:  17.3 y (14.9–19.9 y) | 322 | RPLND OR Surveillance | Pathologic N stage at RPLND  T category  Vascular invasion  Percentage of embryonal  Teratoma | tumour recurrence | multivariable analysis | **Results of recurrence analysis**  Pathologic N stage at RPLND (pN+ vs pN0): OR 2.9 (1.3–6.5)  p=0.009  T category (pT2/3 vs pT1): OR 4.4 (1.7–11.7) p=0.003  Vascular invasion (present vs absent): OR 2.7 (1.2–6.2)  p=0.019  Percentage of embryonal carcinoma (50 vs 6): OR 3.5 (1.4–9.0)  Percentage of embryonal carcinoma (85 vs 6): OR 1.6 (0.6–4.5)  p=0.019  Teratoma (present vs absent): OR 1.1 (0.5–2.4) p=0.891 | LoE 2b  RoB: moderate  no relapse definition |
| Shinoda  2018 | prognostic cohort study  Japan  2005-2008  Median follow-up period: 30.3 mo (0.3-65.6 mo) | 132 (of 159) | Surveillance | Age at orchiectomy  (cutoff greater than 30)  preoperative AFP levels (cutoff: 80 ng/ml)  preoperative LDH level  pT classification  (cutoff greater than 1)  presence of yolk sac  tumor components  embryonal carcinoma elements | distant metastasis  tumor recurrence | chi-squared test or Mann–Whitney U-test  univariate and multivariate analysis | Surveillance group (n= 132)  Risk factors of tumor recurrence  Age at orchiectomy, preoperative AFP levels and LDH level, pT classification and presence of yolk sac  tumor components: no significance  embryonal carcinoma elements: 15 (19.2%)  no embryonal carcinoma elements: 1 (1.8%)  p= 0.0068 (univariate)  p= 0.0252 (multivariate) | LoE 2b  RoB: low |
| Sturgeon 2011 | prognostic cohort study  Canada  1981 – 2005  Median follow-up: 6.3 y (0.08–25.9 y) | 371 | Surveillance | Lymphovascular invasion  presence of pure embryonal carcinoma | Relapse | multivariate analysis | **Risk factors for relapse**  Lymphovascular invasion: HR 3.22 (95% CI, 2.17–4.78), p < 0.0001  Presence of pure embryonal carcinoma: HR 1.74 (95% CI, 1.10–2.74), p=0.02 | LoE 2b  RoB: moderate  prognostic factor is described but not extensively defined |

* Level of evidence was downgraded due to study limitations.

**Abbreviations**

AFP α-fetoprotein

BEP Bleomycin, Etoposide, and Cisplatin

BMI Body Mass Index

DFS Disease-Free Survival

HCG Human Chorionic Gonadotropin

HR Hazard Ratio

IGCCCG International Germ Cell Cancer Collaborative Group

LDH Lactate dehydrogenase

LoE Level of Evidence

NSGCT Nonseminomatous Germ Cell Tumors

OR Odds Ratio

PFS Progression Free Survival

RNA Ribonucleic acid

RoB Risk of Bias

RPLND Retroperitoneal Lymph Node Dissection

RFS Recurrence-Free Survival

USA United States of America

y years
